# Engineered fluorescence for live diagnostics and quantification of poxvirus infection

**DOI:** 10.1099/jgv.0.002311

**Published:** 2026-08-26

**Authors:** Sian Lant, Joe A. Holley, Rebecca P. Sumner, David O. Ulaeto, Carlos Maluquer de Motes

**Affiliations:** 1 Department of Microbial Sciences, Faculty of Health and Medical Sciences, University of Surrey, Guildford GU2 7XH, UK; 2 CBR Division, Defence Science and Technology Laboratory, Salisbury SP4 0JQ, UK

**Keywords:** molecular diagnostics, poxvirus, reporter, reverse transcriptase, vaccinia virus

## Abstract

Global resurgence of human and animal poxviral diseases calls for diagnostic innovation. Here, we explored a novel strategy to quantitate poxvirus infection in living cells through an integrated reporter system specifically activated by infectious particles. Combining constitutive expression of green fluorescence protein (*gfp*) transcripts flanked by poxvirus transcriptional elements and retrovirus reverse transcriptase (RT), we developed a continuous stable cell line where fluorescence was driven by poxvirus transcriptional machinery upon recognition of reverse-transcribed *gfp* transcripts in the cytosol. Constitutively expressed *gfp* transcripts were reverse-complemented to prevent direct translation in the absence of poxvirus infection. Maloney murine leukaemia virus (MMLV) RT was selected for its exclusively cytosolic localization and superior activity. GFP-positive cells were detected only in the presence of reporter cassette and RT and upon infection with vaccinia, cowpox and ectromelia viruses, but not herpes simplex virus, confirming specificity for poxviruses. RNA fluorescent *in situ* hybridization experiments revealed that only cells with high positive-sense *gfp* transcript copy number turned GFP-positive, suggesting a threshold determined by RT activity during poxvirus infection. Nonetheless, positivity was dependent on genome replication, suggesting detection of infectious virus. Our study integrates multiple biotechnological advances to timely pioneer a novel fluorescence-based strategy to diagnose and quantitate virus infection and provides proof-of-concept to produce a reporter gene in its cDNA form in the cytosol of cells, where poxviruses and asfarviruses replication takes place. Subsequent activation by live poxviruses offers distinctive advantages over other detection methods, including the measurement of infectious virus in an automated system with minimal operating requirements.

## Introduction

Poxviruses are a large family of dsDNA viruses infecting a wide range of vertebrate species. Over the last decade, human and animal poxviral diseases have resurged globally. MPXV, a traditionally zoonotic orthopoxvirus (OPXV) endemic to tropical rainforests in West and Central Africa, caused a multi-country outbreak with sustained human-to-human transmission in 2022 and has become a global health challenge [1]. In addition, sporadic OPXV zoonotic events have been reported in Europe [2, 3], Asia [4] and North America [5], and numerous vaccinia virus (VACV) zoonoses in South America are attributed to feral VACV established in wildlife during the smallpox eradication campaign [6]. Besides the global threat to human health, several highly infectious and economically devastating animal poxviral diseases have expanded beyond their original endemic areas. An increasing number of outbreaks of lumpy skin disease have been documented in Europe [7] and Asia [8] since 2015. Similarly, sheep and goat poxviruses have spread into new geographic areas, including traditionally disease-free countries [9, 10]. Their dual mode of transmission (mechanical for short-distance spread; and trading for long-distance, transboundary spread) makes the control of these diseases particularly challenging [10, 11].

The global emergence of poxvirus disease has re-ignited the need for novel therapeutic interventions and diagnostic tools. Quantification of poxvirus infectivity is important not only for diagnosis but also for fundamental research, vaccine production, therapeutics and other biotechnological applications. Quantification is currently achieved through several methods, including cell-based assays and protein or nucleic acid detection. Cell-based assays measure infectivity accurately but are limited because they require appropriate cell substrates and involve relatively long incubation times, particularly with fastidious poxvirus species. Nucleic acid amplification tests and serological assays measure both non-infectious and infectious particles, hence, they lose accuracy to determine infectivity. However, given their speed and cost, PCR-based tests are commonly used to detect poxvirus genomic DNA or early mRNA transcripts, with quantitative PCR most often being used for laboratory diagnosis of OPXV [12–18].

The prototypic virus of the family Poxviridae is VACV, which served as the vaccine to eradicate smallpox and is a model for virus biology. Poxviruses are rare amongst mammalian DNA viruses in completing their entire infectious cycle within the cell cytosol. Contrary to many other viruses where tropism is largely determined by interactions with specific receptors, poxvirus successful replication relies on multiple post-entry events. Upon entry, the viral core is deposited in the cytosol and interacts with the host ubiquitin system to expand and initiate the transcription of early genes [19, 20]. These are released and translated into early viral factors that induce (i) rupture and dissolution of the core wall to mediate release of the dsDNA genome [21, 22] and (ii) evasion of cytosolic immune sensors that can potentially sense the viral genome [23, 24]. Upon genome release, DNA replication takes place forming large viral factories where intermediate and late gene expression also occurs prior to the formation of nascent virions.

The generation of a reporter cell line for the quantification of poxviruses could be a useful tool for diagnostics, therapeutic discovery and fundamental research and offer significant advantages over other methods, including the enumeration of viruses that do not form plaques in traditional cell culture. The use of reporter cell lines for quantifying viruses has been proven to work with a variety of different viruses [25–28]. However, none has been generated for poxviruses. Here, we engineered a stable fluorescent cell line sensitive to poxvirus infection using a combination of molecular tools able to circumvent the limitations posed by their cytosolic replication, providing biotechnological proof of concept for rapid live cell-based detection of nucleo-cytoplasmic large DNA viruses.

## Methods

### Cell culture and viruses

HeLa, HEK293T and the HEK293T-derived reporter cell lines created herein (HEK293TRep and HEK293TRepRT) were grown in Dulbecco’s Modified Eagle Medium (DMEM, Life Technologies) supplemented with 10% heat-inactivated FCS (Biowest), 100 U ml^−1^ penicillin and 100 µg ml^−1^ streptomycin (Pen/Strep, Life Technologies). VACV strains Western Reserve (WR), vv811, modified vaccinia Ankara (MVA), cowpox virus (CPXV) strain Brighton-Red and ectromelia virus (ECTV) strain Moscow have been previously described [29, 30]. VACV.WR expressing A5.GFP [31] and ECTV expressing red fluorescent protein (RFP) [32] were kind gifts from Geoffrey L. Smith and R. Mark Buller, respectively. VACV.F13.mCherry is a derivative of WR with the envelope protein F13 fused to mCherry at the C-terminus and was generated as described before [33]. MVA was grown and titrated in chicken embryo fibroblasts. All other viruses were expanded and titrated in BS-C-40 cells.

### Reverse transcriptase and reporter sequence creation and cloning

The p51 and p66 subunits of the human immunodeficiency virus (HIV) reverse transcriptase (RT) [34] and Maloney murine leukaemia virus (MMLV) RT [35] were codon optimized using the GeneArt Codon Optimization tool online and synthesized (Invitrogen). Each RT sequence was flanked with restriction sites and cloned into an expression vector carrying zeocin resistance and previously modified to express an *N*-terminal FLAG tag [30]. The reporter cassette was also synthesized (Thermo Fisher) and subcloned into a pcDNA4/TO modified to carry puromycin resistance (Addgene). All constructs were verified via Sanger sequencing.

### Generation of HEK293T cell lines

Cell monolayers were grown to 50% confluency in 10 cm dishes and transfected with 5 µg of the required plasmid using polyethylenimine (Merck) in 500 µl of Opti-MEM (Gibco) and incubated at 37 °C for 24 h. The transfected cells were trypsinised and resuspended in the appropriate growth medium supplemented with 2 µg ml^−1^ of puromycin or 100 µg ml^−1^ Zeocin as indicated. The cells were then incubated in 10 cm dish at 37 °C until clonal colonies were visible and expanded individually to generate clonal cell lines.

### SYBR green PCR-enhanced reverse transcriptase assay

Cells grown to 70% confluency in a 6-well plate were transfected with RT for 24 h. Where virus was utilised, this was at a multiplicity of infection (MOI) of 2 plaque-forming units (PFU) per cell for 24 h. After 24 h of incubation at 37 °C, the transfected cells were detached and washed with PBS. Cell dilutions were then created and SYBR green PCR-enhanced reverse transcriptase (SG-PERT) assay was performed as described previously [36] using a LightCycler 96 (Roche). Samples falling outside the standard curve were discounted, and the activity per ml (U ml^−1^) of the RTs tested was calculated by extrapolating the obtained values against the standards of known enzymatic activity (1×10^9^ pU µl^−1^ down to 1×10^2^ pU µl^−1^). Assays were performed in triplicate, and data analysed using the GraphPad Prism v8 software.

### Cellular fractionation

Transfected cells were fractionated using the NE-PER Nuclear and Cytoplasmic Extraction Reagents (Pierce) following the manufacturer’s instructions except for a further PBS washing step added between step 5 and 6 to obtain a cleaner separation of fractions.

### cDNA synthesis and quantitative PCR

All RNA extractions were conducted following the manufacturer’s instructions provided within the Norgen Total RNA Extraction kit with DNase treatment. The resulting RNA concentration was measured on a NanoDrop (Labtech). DNase treatment was performed on column as described by the manufacturer (Norgen DNase on column treatment). Following RNA extraction, cDNA synthesis was performed using Superscript III RT (Invitrogen) as per the manufacturer’s instructions. Subsequently, qPCR was performed on the cDNA generated using fast SYBR green qPCR mastermix (Invitrogen) as outlined in the manufacturer’s protocol. Primers 5′-CATGTGGTCGCGCTTTTCAT-3′ (forward) and 5′-GCGGACCACTATCAGCAGAA-3′ (reverse) were used for detection of reporter construct, whereas primers 5′-ACCCAGAAGACTGTGGATGG-3′ (forward) and 5′-TTCTAGACGGCAGGTCAGGT-3′ (reverse) were used for *GAPDH*. Thermocycling was performed on a LightCycler 96 (Roche). Each reaction was performed in biological triplicate, unless stated otherwise and in technical duplicate. The resulting data were analysed using ΔCt value normalized against the reaction with GAPDH.

### Immunoblotting

Cells were lysed via fractionation (see above) or in radioimmunoprecipitation assay buffer supplemented with 1 U ml^−1^ benzonase (Merck) and denatured at 37 °C in the presence of Laemmli sample buffer. Denatured samples were resolved by SDS-PAGE and transferred to nitrocellulose membranes (GE Healthcare) using a Trans-Blot semi-dry transfer unit (Bio-Rad). Membranes were blocked in PBS supplemented with 0.1% Tween and 5% skimmed milk (Sigma) and subjected to immunoblotting with the following primary antibodies at the indicated dilutions: FLAG (Sigma F1804; 1 : 1,000), β- actin (Proteintech 20536–1-AP; 1 : 5,000), Tubulin (Millipore 94001452; 1 : 5,000). Primary antibodies were detected using IRDye-conjugated secondary antibodies in an Odyssey infrared imager (LI-COR Biosciences).

### Immunofluorescence staining

Cells were seeded on glass coverslips and incubated at 37 °C for 24 h. These cells were infected or transfected as above using PEI (HEK293T cells) or Lipofectamine (HeLa cells) following the manufacturer’s recommendations and then incubated again at 37 °C. After 24 h, immunofluorescence was performed as previously reported [37]. Briefly, the cells were washed twice in PBS and then fixed in 4% paraformaldehyde (PFA) for 20 min. After two further washes in PBS, the cells were quenched in 150 mM ammonium chloride, permeabilized in 0.5% Triton-X-100 and blocked for 60 min in 10% FBS. The cells were stained with primary antibody anti-FLAG (Sigma F1804; 1 : 300) for 60 min, followed by Alexa Fluor 488 goat anti-rabbit secondary antibody (Invitrogen, 1 : 1,000) for 40 min. The cells were washed twice more in PBS, then the coverslips were mounted in Mowiol 4–88 solution containing DAPI. Images were taken on a widefield immunofluorescence microscope.

### Fluorescent *in situ* hybridization

HEK293T cells were seeded on 2-well slide chambers (BioTek) aiming for 50% confluency on the day of fixation. At 24 h post-seeding, the cells were either infected with the required virus at MOI 2 (unless stated otherwise) or transfected with 100 ng of plasmid expressing FLAG-tagged green fluorescence protein (GFP). After a further 24 h, the cells were fixed in 4% PFA. After fixation, the protocols ‘Sample preparation technical note for cultured adherent cells using RNAscope fluorescent assay’ and ‘RNAscope fluorescent multiplex kit user manual, part 2’ (ACD Bio) were followed using probe RNAScope eGFP probe (catalogue number: 400281-C2) (ACD Bio). The coverslips were mounted in Mowiol 4–88 solution containing DAPI. Images were taken on a laser scanning confocal microscope.

### Live fluorescence measurements

Cells were seeded in either 6- or 24-well plate at 3×10^5^ cells ml^−1^ and infected at MOI 2 (unless stated otherwise) for 24 h at 37 °C (unless stated otherwise). The cells were then analysed using a Cytation 5 fluorescent plate reader using the 4× objective with auto-focus. The background was minimized by setting the exposure. Brightfield and GFP were scanned, alongside RFP if using a fluorescently tagged virus. After scanning, the image was pre-processed using Gen5 software version 13 and deconvoluted. For 6-well plate image analysis, four images at the same location in each well (avoiding images at the periphery of the well) were analysed via ImageJ software for a GFP-positive cell count. The threshold was set to remove background. The perimeter of the well was excluded from the analysis to avoid including cells falsely identified by the camera as expressing high levels of GFP. For 24-well plate analysis, the images of the well were stitched with 16% size reduction and scanned for GFP-positive cells. The brightfield images were used to discern cells from debris. To exclude background signal and non-cell entities, masking was performed to only include GFP fluorescent levels of over 8000 and objects between 10–100 µm in size. The experiments were performed in triplicate unless stated otherwise. Student’s t-test with Welch’s correction was performed via GraphPad Prism v8 software as indicated in the figure legends.

## Results and discussion

### Design of a poxvirus reporter gene system

**Fig. 1.**
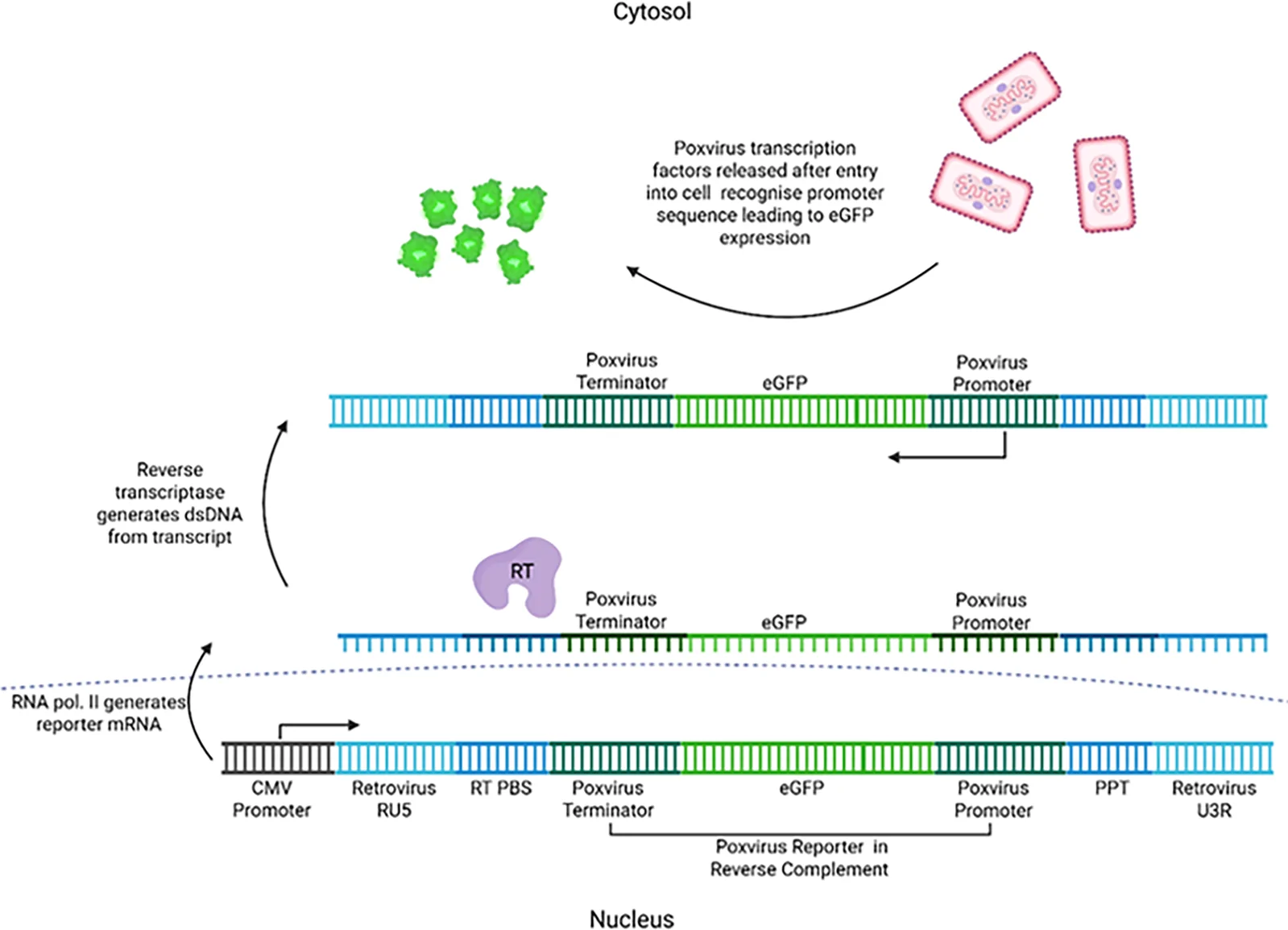
Design of a poxvirus reporter gene system. [1] A reporter cassette consisting of the poxvirus early/late promoter, eGFP and poxvirus terminator (grey) is flanked by the retrovirus RT elements (yellow). The construct is under the control of the human CMV promoter (orange) [2]. RNA Pol II drives synthesis of reporter RNA which contains retrovirus elements for recognition by ectopic RT and synthesis of dsDNA [3]. Upon poxvirus infection, reverse-transcribed DNA containing the gfp transcript is transcribed and translated into eGFP, resulting in fluorescent cells. Image generated with BioRender^®^.

The poxvirus replication and expression machinery operates in the cell cytosol, segregated from the nucleus, but can mediate the replication and expression of heterologous plasmids and DNA molecules in trans [38]. Reporter genes in the cell nucleus are, therefore, not inducible by poxvirus infection. However, if converted into cDNA in the cell cytosol, poxvirus infection could mediate their expression and translation in a manner suitable for rapid and sensitive detection. Conversion to cDNA could be accomplished by RTs. To explore this principle, we designed a cassette containing a reporter gene flanked with the strong synthetic poxvirus pE/L promoter and a terminator (Fig. 1). The enhanced green fluorescent protein (eGFP) was chosen as the reporter due to its brightness, stability and extensive use in research settings, with restriction sites offering the option of generating a luciferase-based reporter cell line in future for higher sensitivity bulk quantitative analysis. The poxvirus cassette was flanked by the long terminal repeats (RU5 and U3R) containing the retrovirus RT elements, RT primer binding site and a polypurine tract, all required for RT activity. To avoid spontaneous translation of the reporter gene in the absence of infection, we initially envisaged using the promoter and terminator of the human RNA polymerase I (RNA Pol I), which does not produce protein-encoding RNA. However, the RNA Pol I promoter is weaker than RNA Pol II [39, 40]. Hence, the complete cassette was cloned downstream of the minimal cytomegalovirus (CMV) promoter under the control of RNA Pol II and the poxvirus cassette was included in its reverse complementary sequence.

### Expression, localization and activity of RT enzymes

**Fig. 2.**
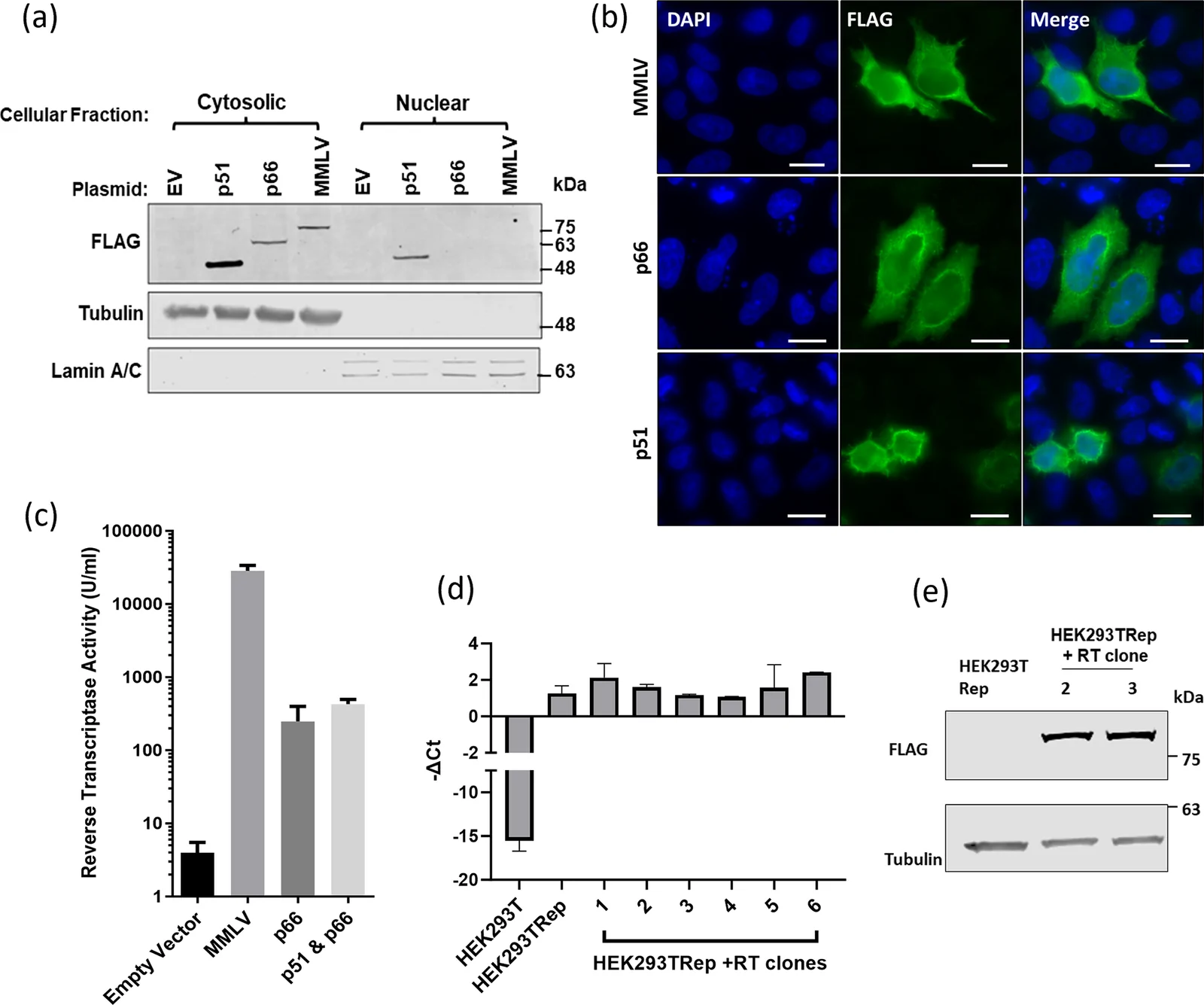
Expression, localization, and activity of FLAG-tagged MMLV, p51, and p66 RT, and stable expression confirmed in HEK293TRep clones. (**a**) Cell fractionation showing localization of transfected FLAG-tagged RT enzymes in HeLa cells. (**b**) Fluorescence microscopy of HeLa cells transfected for 24 h with MMLV RT and the HIV RT subunits, p51 and p66. Cells were fixed and immunostained for FLAG-tagged RT (green) and subsequently mounted in the presence of DAPI (blue). Scale 10 µm. Images are representative of three experiments. (**c**) Empty vector (EV), MMLV RT, p66 HIV RT subunit (alone or in combination with the p51 subunit) were transfected into HEK293T cells for 24 h. Cells were then subjected to SG-PERT assay followed by qPCR with a standard curve for absolute quantification. The experiment was performed in duplicate and is representative of two repeats. Data shown as the mean±sem. (**d**) RT qPCR showing change in Ct of reporter vs *gapdh* in HEK293T, HEK293TRep and HEK293TRepRT cells after clonal selection. The experiment was performed in triplicate and is representative of two repeats. Data are shown as the mean±sem. (**e**) Western blot analysis of HEK293TRepRT clones vs parental HEK293TRep cells to investigate FLAG-tagged MMLV RT expression. Membranes were probed for Tubulin (loading control) and FLAG.

RT is a retrovirus enzyme packaged within the capsid that converts the viral RNA into cDNA before integration. Recombinant RT enzymes are also used in reverse transcriptase PCR (RT-PCR) reactions. We were unaware of reports on the use of RT enzymes in isolation after ectopic delivery in cells. We selected the RT from the MMLV, a monomeric enzyme and the HIV, which contains a catalytic subunit termed p66 and a structural subunit termed p51 [41]. These RT genes were codon-optimized for expression in human cells and cloned into a previously modified pcDNA4/TO vector containing three copies of the FLAG epitope sequence in its N-terminus [30]. The FLAG-tagged RT plasmids were transfected into HeLa cells and expression and localization of the encoded proteins were analysed. Firstly, lysed transfected cells were separated into cytosolic and nuclear fractions. Immunoblotting for FLAG revealed strong bands for all RT molecules that were absent in the mock-transfected control sample (Fig. 2a). These bands were detectable in the cytosolic fraction and absent in the nuclear fraction, with the exception of the HIV subunit p51 that showed detectable expression in the nucleus. The samples were also blotted for the cytosolic protein α-tubulin and the nuclear protein Lamin A/C as controls for correct fractionation (Fig. 2a). To confirm that RT localized within the cytosol, immunofluorescence was performed. HeLa cells were transfected with each RT enzymes for 24 h and subsequently fixed and immunostained for FLAG. RTs were distributed diffusely in the cytosol, but not in the nucleus, of transfected cells (Fig. 2b).

To assess that ectopically transfected RT remained functional, we adapted a SG-PERT assay that uses genomic RNA from the bacteriophage MS2 as a substrate, which is commonly used to quantify retrovirus RT activity [36, 42]. HEK293T cells were transfected with either empty vector (EV), MMLV, p66 or p51 combined with p66. After 24 h, SG-PERT assay was performed and activity for each RT was calculated from the standard curve. Whilst minimal activity was observed in cells transfected with EV (4 U ml^−1^), high levels of activity were found for the MMLV RT, with an average of 28,467 U ml^−1^ (Fig. 2c). Both p66 on its own and in combination with p51 showed lower activity, measured at 249 and 428 U ml^−1^, respectively. Collectively, the results indicated that high levels of expression and activity could be obtained using the RT from MMLV, and hence this enzyme was taken forward.

To generate a stable reporter cell line, HEK293T cells were chosen due to their high transfection efficiency and protein production capacity. HEK293T cells are also defective for cytosolic DNA sensing unless the stimulator of interferon genes is reconstituted [37, 43], hence avoiding the potential recognition of reporter cDNA produced by RT. HEK293T cells were transfected with the reporter plasmid and subjected to selection with puromycin until individual clones were visible and subsequently expanded. To determine the level of reporter RNA expressed in these cells, RT-qPCR was performed using primers designed to span the central section of the reporter and normalized against *gapdh*. Multiple clonal cell lines produced low Ct values indicative of high expression, one of which was selected and is shown in Fig. 2d. A clonal HEK293T cell line containing the reporter construct (HEK293TRep) was then transfected with the plasmid expressing MMLV RT and multiple clonal cell lines were selected under zeocin resistance (HEK293TRepRT). RT-qPCR analysis of these clones demonstrated that RT transfection did not affect the levels of reporter expression, with all clones showing a similar ΔCt to the parental HEK293TRep cells. Detection was specific, as no signal was detected in untransfected HEK293T cells, and data were presented as -ΔCt (reporter minus *gapdh*) (Fig. 2d). Two of these clones were subjected to immunoblotting to confirm expression of MMLV RT enzyme (Fig. 2e). We also confirmed that all cells within the clonal population expressed RT, with immunofluorescence images showing similar cytosolic diffuse staining as in Fig. 2b (data not shown).

### Detection of poxvirus infection via reporter gene cell line

**Fig. 3.**
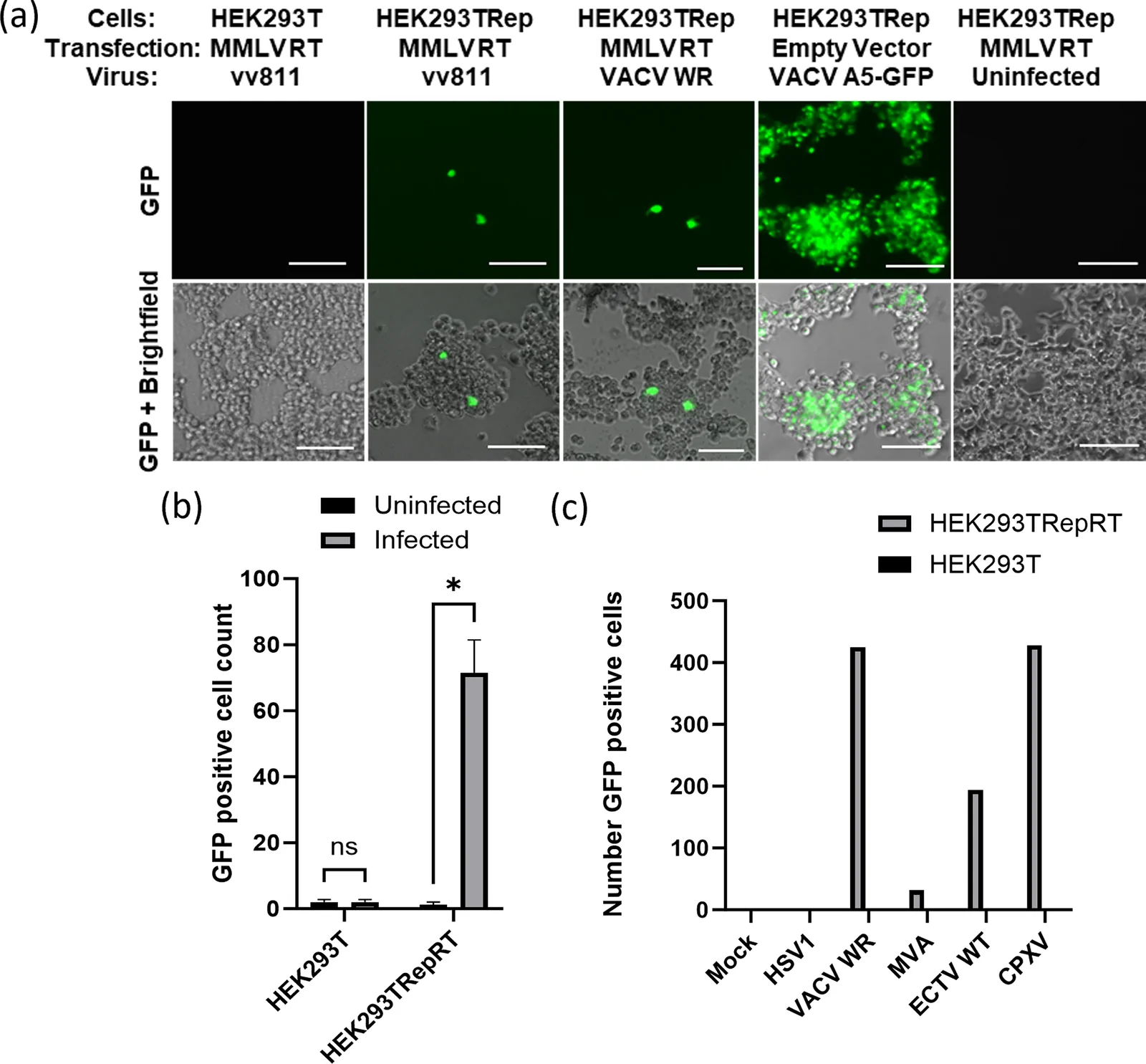
VACV infection of cells expressing reporter and RT. (**a**) Cells were either mock-transfected or transfected with MMLV RT for 24 h. Subsequently, the cells were either mock-infected or infected with VACV vv811 or WR at MOI 2 for 24 h. Only HEK293TRep (but not HEK293T) cells transfected with MMLV RT and infected with vv811 or WR (but not mock-infected) yielded GFP-positive cells. Infection with VACV A5-GFP at MOI 2 was included as a further control. Scale 50 µm. (**b**) HEK293T and HEK293TRepRT cells were infected for 24 h MOI 2 vv811 and visualized using a Cytation5 fluorescent plate reader. GFP-positive cells were counted in randomly selected unstitched images. (**c**) HEK293T and HEK293TRepRT cells were infected as above with the indicated viruses. Data are shown as the mean of four images ±sem. Unpaired non-parametric Student’s t-test with Welch’s correction was applied for statistical significance. ns, non-significant; *P<0.05.

To test the functionality of the system, we used prototypic VACV WR as well as vv811, an attenuated VACV mutant lacking 55 open reading frames [44, 45]. Infection of HEK293TRep cells carrying RT (HEK293TRepRT) with either vv811 or WR generated some GFP-positive cells and this was not observed in control conditions where HEK293TRep cells were infected in the absence of RT or in uninfected HEK293TRep carrying RT (Fig. 3a). This indicated that fluorescence was not caused by spontaneous *gfp* translation in the absence of poxvirus infection or autofluorescence induced by viral cytopathic effect (CPE). Quantification via an automated cell imaging reader determined that ~80 cells turned GFP-positive in a field of ~1,000, which represented ~0.1% of the total cell population (Fig. 3b). Although this positivity was low in a high multiplicity infection, specificity remained high when we repeated this experiment using different OPXV species as well as the unrelated, nuclear-replicating dsDNA virus herpes simplex virus 1 (HSV1). Reporter cells infected with HSV1 did not show GFP-positive cells, whereas these were recorded in OPXV infections, including MVA, ECTV and CPXV (Fig. 3c).

### Positive-sense *gfp* fluorescent *in situ* hybridization and RT activity in VACV-infected cells

**Fig. 4.**
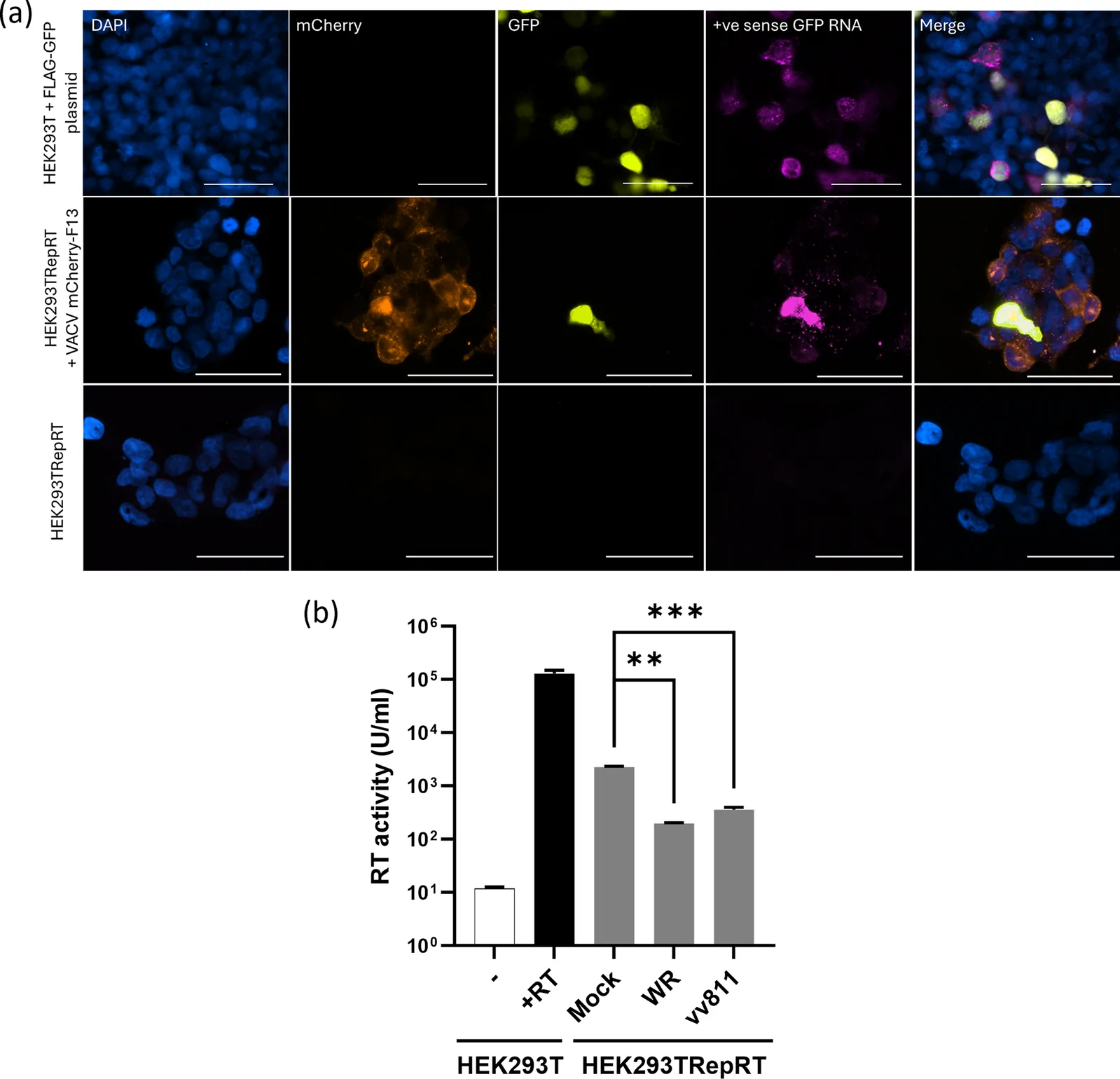
Positive-sense *gfp* FISH and RT activity in VACV-infected HEK293TRepRT cells. (**a**) FISH probes against positive-sense *gfp* mRNA were used on HEK293TRepRT cells that were either uninfected or infected with WR.F13.mCherry at MOI 10 for 24 h. HEK293T cells transfected for 24 h with a plasmid expressing GFP were included as a further control. Scale 10 µm. The experiment is representative of three experiments. (**b**) SG-PERT was performed on HEK293T cells mock-transfected or transfected with 1 µg MMLV RT as well as on HEK293TRepRT cells mock-infected or infected with VACV WR or vv811 at MOI 2 for 24 h. The experiments were performed in biological triplicate and technical duplicate. Data are shown as the mean±sem. An unpaired Student’s t-test was applied for statistical significance. **P<0.01, ***P<0.001.

To establish whether the absence of GFP in the majority of infected cells was due to reporter transcription being absent or under the limit of detection, RNA fluorescent *in situ* hybridization (FISH) was applied using fluorescent probes specifically designed to hybridise with positive-sense *gfp* mRNA. RNA FISH was performed on HEK293TRep cells infected with VACV for 24 h or mock-infected, alongside HEK293T cells transfected with a plasmid expressing GFP for use as a control. A VACV expressing mCherry was used to monitor infectivity. As expected, uninfected HEK293TRepRT cells were negative for positive-sense *gfp* mRNA, whereas transfected cells showed detectable amounts of *gfp* mRNA that resulted in diffuse GFP fluorescence (Fig. 4a). On the contrary, HEK293TRepRT cells infected with VACV were largely negative for *gfp* mRNA despite showing mCherry expression. Detectable levels of GFP were only observed in a few cells and this correlated with *gfp* mRNA detection (Fig. 4a). A further SG-PERT assay was performed to assess whether RT activity from the constitutively expressing cell line differed from transient transfection and was affected by poxvirus infection. The activity of the RT appeared roughly 100-fold less in the stable cell line as compared to transiently transfected cells, with infection inducing a further reduction in RT activity (Fig. 4b). Collectively, these results indicated that despite all cells having all the components of the system and being infected effectively, RT activity mediating production of positive-sense *gfp* transcripts was not sufficient in the majority of cells to yield detectable levels of GFP protein upon infection.

### Dose-dependent reporter activation

**Fig. 5.**
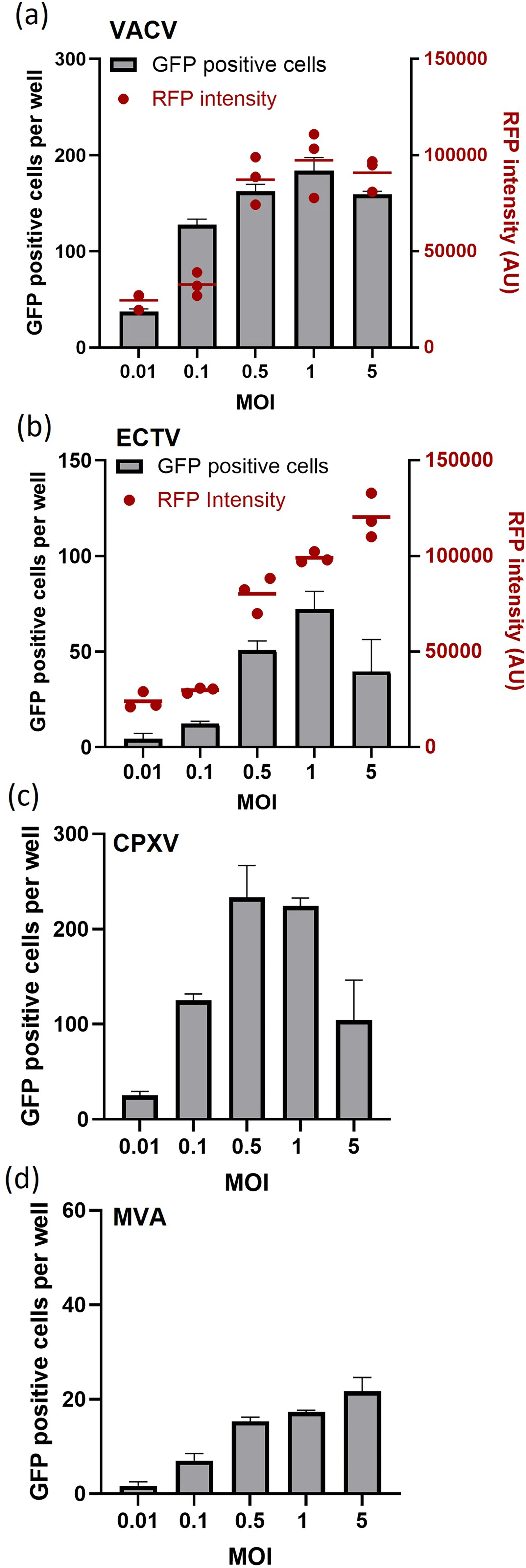
Dose-dependent reporter activation by OPXV infection of HEK293TRepRT cells. Cells were infected at the indicated MOI for 24 h with (**a**) VACV.mCherry.F13, (**b**) ECTV.RFP, (**c**) CPXV or (**d**) MVA. GFP-positive cells were counted and, if applicable, mCherry/RFP intensity was measured via a Cytation5 fluorescent plate reader. The experiment was performed in triplicate and is representative of at least two experiments. Data are shown as the mean±sem.

To understand the dynamics of GFP generation in our reporter cell line, we recorded GFP positivity following dose-dependent infections with different OPXV. A wide range of multiplicities of infection (MOI) from 0.1 to 5 p.f.u./cell were employed using VACV.mCherry and ECTV.RFP. Detection of mCherry/RFP allowed simultaneous monitoring of infection levels. GFP positivity increased in a dose-dependent manner for both viruses, reaching maximal positivity around MOI 1 (Fig. 5a, b). Higher MOI resulted in fewer GFP-positive cells, suggesting detrimental effects on RT activity as observed before. At MOI 1, VACV infection yielded ~200 GFP-positive cells, which would represent roughly 0.1% of the total cell population. At the same MOI, ECTV.RFP infection yielded lower positivity (~70 GFP-positive cells). We also tested CPXV at this range of MOI and observed similar results (Fig. 5c). We then utilized our reporter cell line to measure infection by the highly attenuated vaccine strain MVA. MVA was selected after serial passage in chicken cells and is unable to replicate in most mammalian cell lines due to a post-replicative defect. MVA infections yielded fewer GFP-positive cells (~20 cells, 0.01% of the total population) (Fig. 5d), a marked decrease compared to replicative VACV.

### Replication-dependent reporter activation

**Fig. 6.**
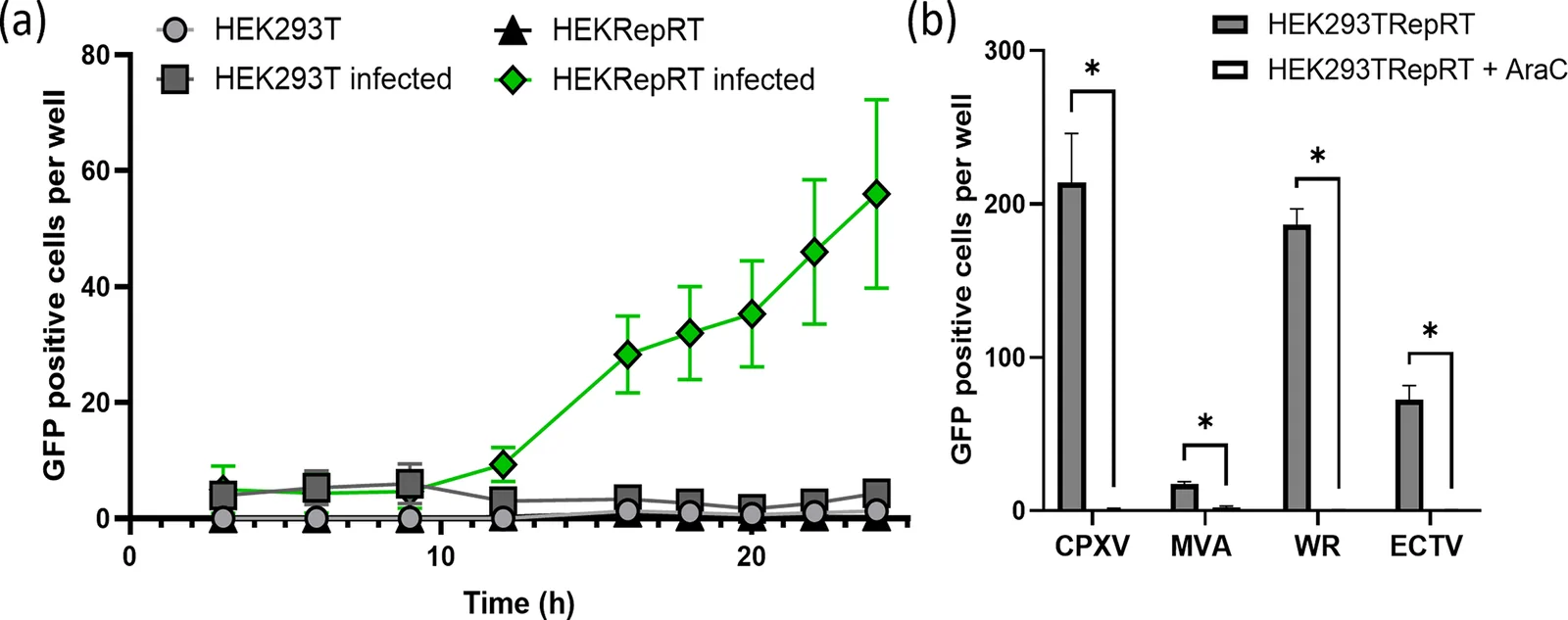
Time- and replication-dependent reporter activation. (**a**) HEK293TRepRT cells were mock-infected or infected with VACV 811 at MOI 2 and GFP-positive cells were measured at multiple times post-infection using a Cytation5 fluorescent plate reader. (**b**) HEK293TRepRT cells were infected with a variety of OPXV for 24 h in the presence or absence of AraC. GFP-positive cells were subsequently measured using a Cytation5 fluorescent plate reader. The experiments were performed in triplicate and are representative of at least two repeats. Data are shown as the mean±sem. Unpaired non-parametric Student’s t-test with Welch’s correction was used for statistical significance. ns, non-significant; *P<0.05.

Data from MVA infections suggested that late gene expression significantly enhanced reporter activation. To investigate the temporal aspects of the reporter cell line, reporter cells were infected and GFP production measured over a period of 24 h (Fig. 6a). GFP levels became only detectable at 16 h post-infection, supporting the notion that late gene expression and a complete replication cycle triggered stronger reporter activation. To investigate this, HEK293TRepRT cells were infected with MOI 1 of VACV WR, ECTV, CPXV and MVA in the presence or absence of 40 µg ml^−1^ of cytosine arabinoside (AraC), a commonly used inhibitor of DNA replication. All viruses activated the reporter system as observed in previous infections. However, this was completely abolished in the presence of AraC (Fig. 6b). These data indicated that activation of the reporter system was significantly enhanced by post-replicative events in the poxvirus lifecycle. Furthermore, in reporter-activated cells, infection had reached genome replication and late gene expression, consistent with detection of productive rather than abortive infection.

### Usability of the cell line

The OPXV genus includes the human pathogens VARV and MPXV, zoonotic species like CPXV and species used as vaccines like VACV and MVA. The recent emergence of MPXV as a global human pathogen has highlighted the need for novel and more accurate diagnostic methods to detect and study OPXV. Surveillance of OPXV, including MPXV, is likely to require a combination of molecular [46] and antigen tests [47, 48] that can be deployed in endemic and non-endemic countries. Although these detection methods do not discriminate between infectious and non-infectious material, they are rapid, cost-effective and do not require skilled operators. Here, we describe a prototype of a rapid cell-based diagnostic tool for the specific quantification of OPXV infectivity. The system operates on the basis of generating cytosolic DNA complementary to a reporter gene encoded within susceptible cells. We demonstrate that this process can be carried out by retrovirus RT delivered in isolation. It is conceivable that this step is rate-limiting, due to the intrinsic stability and activity of RT outwith the context of infection and the half-life of the DNA products in the cell cytosol. Indeed, retroviral reverse transcription normally occurs in a structured ribonucleoprotein environment and may depend on features and cofactors not reproduced by expressing MMLV RT alone. These elements might be especially relevant for the efficient conversion of the reporter RNA in cells. Additionally, OPXV are known to induce potent host shut-off which likely resulted in further reduction of RT activity. A further possibility is that barrier-to-autointegration factor (BAF), or an analogous protein, binds cytosolic DNA and interferes with the reporter. Even if the poxvirus B1 kinase inactivates BAF [49, 50], this might not be sufficient to relieve BAF from bound reporter molecules or only occur late during infection. In the future, single-cell analyses of reporter RNA and complementary DNA levels, combined with markers of viral infection, would provide valuable insights into the reasons underlying the low proportion of GFP-positive cells. Such analyses would be particularly informative if performed in a high-throughput manner to enable the profiling of sufficiently large numbers of cells to identify and characterize the population of cells that ultimately turn positive.

Our reporter cell line appeared to be specific for poxviruses. In addition, there were no GFP-positive cells in the mock or infected cells lacking the reporter construct. Therefore, there is little chance of false positives occurring with this technique. Due to the nature of the reporter requiring infected cells to be read via an automated system, there is also little room for human error. The system appears to be utilizing the late elements in the reporter, which is in line with early gene expression being largely driven from within the viral core. As uncoating and genome release license expression of post-replicative genes, the cell line appears to be detecting virions that successfully penetrate and uncoat in the cell cytosol, which can be considered a good proxy for infectivity. Although we did not specifically test the limit of detection of our tool, we established a positivity rate of ~0.1%. At MOI 0.01, which is around 4×10^3^ p.f.u. in the context of the assay used, around 50 cells were GFP-positive per well (Fig. 5a). This number is in line with WHO guidelines for mpox diagnostic tools [51, 52] . Available from: https://www.who.int/publications/i/item/9789240076464). Altogether, these observations indicate that further iterations of this prototypic cell line could become a functional tool for diagnosing infectious poxviruses, offering distinct and complementary advantages over other diagnostic methods.

## Conclusion

Herein, we have described the generation of a reporter cell line that utilises ectopically expressed RT for the detection of OPXV. The HEK293T-based reporter cell line can detect infectious poxviruses (at a limit established at 5,000 p.f.u. or higher) within 24 h of infection requiring minimal reagents and manipulation and producing no false positives. Optimization of RT activity to maximize poxvirus-translatable reporter copy number is likely to be critical to enhance performance. The cell line offers advantages over current detection methods, requiring limited technical knowledge, no primer design and few reagents and can be adapted as a valuable diagnostic tool for the detection of live OPXV, including attenuated strains.

## Abbreviations

AraC: cytosine arabinoside
BAF: barrier-to-autointegration factor
CMV: cytomegalovirus
CPE: cytopathic effect
CPXV: cowpox virus
ECTV: ectromelia virus
EV: empty vector
FISH: fluorescent *in situ* hybridization
GFP: green fluorescence protein
HIV: human immunodeficiency virus
HSV1: herpes simplex virus 1
MMLV: Maloney murine leukaemia virus
MOI: multiplicity of infection
MVA: modified vaccinia Ankara
OPXV: orthopoxvirus
PFA: paraformaldehyde
PFU: plaque-forming units
RFP: red fluorescent protein
RNA Pol 1: RNA polymerase I
RT: reverse transcriptase
RT-PCR: reverse transcriptase PCR
SG-PERT: SYBR green PCR-enhanced reverse transcriptase
VACV: vaccinia virus
WR: Western Reserve
